# A review of supply chain quality management practices in sustainable food networks

**DOI:** 10.1016/j.heliyon.2023.e21179

**Published:** 2023-10-26

**Authors:** Patrick Robert Burgess, Funlade T. Sunmola, Sigrid Wertheim-Heck

**Affiliations:** a Department of International Studies and Consultancy, Aeres University of Applied Sciences, Dronten, the Netherlands; b School of Physics, Engineering, and Computer Science, University of Hertfordshire, Hatfield, United Kingdom; c Environmental Policy Group, Wageningen University, Wageningen, the Netherlands

**Keywords:** Short food supply chain, supply chain management, Quality management, Local food

## Abstract

Supply chain quality management practices are necessary to improve processes, meet consumer quality needs, and enhance supply chain quality management performance in sustainable food networks. Food supply chain quality management and associated practices are considerably studied in global food systems, less so for alternative food networks. There are salient differences between global food systems and alternative food networks, which may reflect on the applicable supply chain quality management practices in the food systems and networks. This paper reviews the literature on supply chain quality management practices, with a focus on alternative food networks. A systematic literature review methodology is adopted, resulting in the analysis of seventy-eight papers, identifying a total of one hundred and three supply chain quality management practices. The identified supply chain quality management practices were analysed in relation to their link to a) place, production, and producer and b) link to (bio)processes. Emerging themes from the analysis are discussed, and some areas of future research were put forward.

## 1 Introduction

Supply chain quality management (SCQM) has emerged from combining supply chain management and quality management [1], shifting from an internal organisational view of quality management to a supply chain-wide perspective. Supply chain quality management is the coordination and integration of all supply chain activities and stakeholders to monitor, analyse, and continually improve services, processes, and products, leading to value addition to meet the needs of consumers [2]. There are many benefits of SCQM, including enhanced supply chain integration, improved customer satisfaction, improved organisational performance, and improved supply chain performance [3,4]. SCQM aims to improve quality performance, integrate supply chain members, and enable consumer-driven value addition created through upstream and downstream linkages in the supply chain [5,6].

Supply chain quality management is indispensable to the food industry [7]. Food companies can improve their performance by adopting SCQM [8], arising from, for example, increased food safety, improved reputation, enhanced recall procedures, reduced quality risks, and improved consumer quality perceptions [9]. The need for food SCQM spans across food systems. A food system refers to all people and activities required to grow, transport, and consume food [10], encompassing networks of food supply chains. A food supply chain is a series of actors, processes, and operational activities, taking food from a raw material state to a value-added product to meet the end consumer's needs [11]. In such supply chains, consumer trust, particularly regarding quality, is paramount. However, it is increasingly becoming clear that consumer trust differs across networks of supply chains in food systems [12]. For example, consumer trust for quality in the supply chain of global food systems has become impacted on due to intentional and unintentional food incidents, like food fraud, foodborne pathogens, and quality risks [13]. In contrast, alternative food systems (AFS) are increasingly being trusted by consumers due to direct interaction and close relationships between stakeholders [14]. An alternative food system is built to address the issues faced in global food systems and is driven by consumer quality and sustainability needs [15].

Alternative food systems incorporate initiatives that aim to restructure their organisation and supply chain through reduced physical and social distances. These initiatives are commonly referred to as alternative food networks (AFN) [16,17]. The emergence of AFNs dates back to the 1960s when a movement to (re) localise (i.e. moving back from global to local food systems) food consumption and production had occurred [18]. AFNs use alternative and sustainable food supply chain practices that are developed to offset the impact of supply chains in global, industrialised food systems [19], particularly regarding sustainability in AFNs, i.e. reduced food miles, improved ecological production methods, fair value for all stakeholders, and improved relationships [20].

Within AFNs, quality and sustainability are key contributors to value addition [21], and are increasingly being studied. Quality in AFNs has been addressed concerning consumer preferences [22], consumer motivation [23], consumer satisfaction [24], transparency [25], and sustainability [17,[26], [27], [28]]. The characteristics of AFNs lead to differences in quality compared to the supply chain networks in global food systems. SCQM at global levels is mainly developed according to industrialised quality systems like GlobalGap, International Standards Organisation (ISO), and International Food Standard (IFS), and may be challenging to implement in AFNs, especially in small-medium enterprises (SMEs) [29]. SMEs are often found in the AFNs, and less so are large organisations [30]. Quality in AFNs is based on the (re)connection between consumption and production and (re)establishing trust, resulting in AFN initiatives that may fall outside the scope of institutionalised food quality management systems. Falling outside these quality management systems may lead to barriers such as a) market entry and b) potential for non-compliance when using more informal distribution channels, such as a local farm market [29]. The differences in the requirements for SCQM between AFN in the alternative food systems and the supply chain networks in global food systems also have some bearing on the SCQM practices adopted.

Supply chain quality management practices are a set of activities and processes adopted to achieve quality goals from producers to consumers [31]. The literature identifies a variety of SCQM practices that apply to supply chains in general. They include quality leadership (top-level management), customer focus, IT-enabled organisation, supply chain integration, quality management, customer quality involvement, information sharing, cooperation, and continuous improvement [32,33]. These practices have been highlighted in literature reviews on SCQM practices for mainstream supply chains in global food systems, e.g. SCQM practices in food manufacturing [34], and SCQM management practices in fresh and perishable food supply chains [35]. Literature is emerging regarding SCQM in AFNs, illustrating the importance of quality management, quality controls, and processes for stakeholders in AFNs [36], and enhanced performance and conformance through quality controls and assurances [37].

Identifying relevant practices is important in facilitating an appropriate understanding of SCQM in and between different food systems and networks and enabling improved levels of supply chain performance. Until now, research has mainly specified the SCQM management in supply chains that are adopting top-down, supply-driven approaches (i.e. global food systems), while research is limited regarding food supply chains that are designed on a value-driven, bottom-up approach, where quality is defined from the consumer side (i.e. alternative food network). This paper contributes to the literature on SCQM practices focusing on alternative food networks, applying a systematic literature review methodology to establish from the literature the SCQM practices associated with AFNs. The research aims to provide answers to the following research questions: RQ1: What are the supply chain quality management practices in alternative food networks? RQ2: How do the supply chain quality management practices link to quality conventions in alternative food networks?

Section 2 contains an overview of sustainable food networks, emphasising AFNs and insights into SCQM practices in global food supply chains. The research methodology adopted in this paper centres on systematic literature review (SLR) and is presented in Section 3. The results of the SLR conducted are presented in Section 4. The emerging themes observed from the SLR are contained in Section 5, followed in Section 6 by a discussion of the results and a proposal for follow-up research. The paper ends in Section 7 with conclusions.

## 2 Background

### *2.1* Supply chain design in alternative food networks

Sustainable food networks aim to deliver value to stakeholders while upholding social, environmental, and economic activities, processes, and outcomes [17]. Within sustainable food networks, some initiatives, such as AFNs, are looking towards offering sustainable alternative products compared to those in more global food systems [38]. Food supply chains in the global food systems and alternative food networks differ by design, where value can be defined by either a demand-driven or commodity-driven approach [39]. Commodity-driven supply chains focus on cost reduction, increased margins, efficiency, and improved market share. Consumer-driven supply chains are based on differentiation, relationships, transparency, communication and fair profit sharing among stakeholders [40].

The consumer-driven supply chain characteristics are found in many AFNs. In consumer-driven chains, producer relations develop strategies to create value, socio-technical innovations, and producer associations. At the consumer level, there is a desire to understand the product's origin and establish provenance. Processing and retailing stakeholders are locally based, differ in size, scale, and offerings, are focused on quality, and are built to support transparency. Institutional frameworks are more locally oriented, where a local authority is involved and has lower levels of bureaucracy. Associational frameworks are relational and trust-based, formulated regionally, and can also be collaborative [41].

The types of AFNs defined by Ref. [42] provide an understanding of how supply chains are structured in AFNs. The AFN types are a) F*ace-to-face (direct supply chain)*, where consumers and producers interact directly, and consumers buy directly from the producer. Information flow, authenticity and trust in this type are facilitated through direct interaction; b) *Spatial proximity,* where products are produced, processed, and retailed in a specific region. In this scenario, the consumer is made aware of ‘local’ by the point of sales. This category also includes sales through restaurants, pubs, hospitals, schools, care homes and prisons; and c) *Spatially extended* refers to when information about the product and processes is provided to consumers outside the production region using labelling, certification, and branding. There are many initiatives within the three types of AFNs, and their diversity is notable. Some examples of the AFN initiatives fall under short food supply chains, local food systems, regional food supply chains, farmer markets, e-commerce/direct channels, organic food supply chains, farm shops and markets, urban agriculture, box schemes, fair trade, and community-supported agriculture [17,43,44]. These AFNs are value-driven and involve sustainable food entrepreneurs who develop their organisations through experience and aim to professionalise operations and practices to meet future business ambitions [45].

There are some significant differences between global food supply chains and alternative food supply chains (i.e. the supply chains in AFNs). In particular, the supply chains AFNs aim to reduce social and physical distances between producers and consumers, have high levels of supply chain integration, higher levels of transparency and more fair value distribution, and a reliance on trust instead of structural information communication [44,46]. Other key differences include the type of assurance systems employed (i.e. third-party vs. socially monitored), the size of actors in the supply chain, where the economies of scale benefit larger organisations in global supply chains, and decision power across the supply chain [46]. Supply chains in AFNs show a positive relationship with economic and social performance in terms of creating a fairer price for producers and ethical farming practices. Global food supply chains benefit from more well-developed and efficient transport networks, improving environmental performance [47]. The practices employed in the supply chains also result in substantial differences, where a practice that supports the supply chains in global food systems may act as a barrier for those in AFNs, driving a need for understanding the practices. The development of SCQM practices can support these ambitions through, for example, increasing trust and the ability to meet quality requirements for improved access to markets.

### 2.2 Quality conventions in alternative food networks

Alternative food networks are defined in several ways, some of which are used to reduce the distance between producer and consumer, support smaller farm/organisation size, use of holistic or organic production methods, use of local sales channels and cooperatives, and support commitment to the triple bottom line of sustainability [48]. The alternative and sustainable supply chain practices (i.e. organic, fair trade, and proving the designation of origin) in AFNs endeavour to give consumers a substitute choice compared to the offerings of supply chains in the global food systems [17,19]. Supply chains in AFNs are often short and reflect the desire to reduce physical and social distances between buyers and producers [42]. The links between stakeholders are also important, as close and direct supply chain relationships are fundamental to AFNs [16]. In addition to the close linkages, the supply chain in AFNs focuses on enhanced levels of the three-bottom line of sustainability [17]. The three-bottom line in the AFNs relates to the need for fair economic returns for buyers and sellers, social responsibility, and ecologically responsible production and distribution [17]. The literature suggests that the AFN involves stakeholders who desire to provide offerings outside the supply chains in global food systems [17].

AFNs are built on practices for food provisioning that differ from those in global food systems [49]. AFNs are usually grassroots organisations that work towards re-organising the agri-food sector, focusing on one or multiple sustainability pillars (economic, society, environment) [50]. Quality is difficult to define in AFNs. AFNs specify quality from the consumer end of the supply chain, as [50] express several quality drivers in AFNs, including commercial (price and value), industrial (compliance with standards), domestic (trust and traditional production), public (trademarks and brands), inspirational (value conveyed by-product), and technological. [50] also discuss the role of hard quality (i.e. price, standards, trademarks) and soft quality (tradition, environment and community, trust, and community) and highlight the importance of soft quality within the alternative food networks. Alternative food networks have been conceptualised to capture a range of sustainable food transitions, including, for example, (re) localisation (bringing food from global to local), short food supply chains (reduced physical and social distances), and sustainable production methods (organic). Alternative food networks often cover a broader context to support an alternative economic space that opposes the more comprehensive, global food system approach. The alternative food network approach can foster regional economies by supporting technological, organisational, and territorial transformation [41], indicating a need for socio-technical innovations.

Quality is a central theme in the AFNs [51]. Unlike quality in the food supply chains in global food systems defined by multinational players (i.e. supermarkets) and governmental institutions [51], quality in the AFN is consumer-driven. It reflects on consumer perceptions of freshness, taste, and fair value. AFNs also link to quality through organic production, direct sales channels, and protected denomination of origin [17]. The norms value and standards have been referred to in literature as the quality conventions, and they fall under two main categories a) link to place, production, and producer, and b) ecological (link to bioprocesses) [44], see Table 1.

**Table 1:** Quality conventions in AFNs*.

Link to place, production, and producer	Link to bioprocesses
Designation of origin	Integrated
Cottage and farm foods	Organic
Speciality	Natural
*Typical	Healthy and safe
On-farm processed	GMO-Free
Fair Trade/Fair Price	Safe
*Traditional	Free-range
Fresh	
Seasonality	

Source [42,44]: *Typical (Speciality products specific to a region or place of production). *Traditional (production and processing methods specific to regions and products).

The quality conventions in Table 1 are connected to quality and sustainability in the supply chain of AFNs. AFNs aim to achieve several levels of sustainability. Economic outcomes focus on producers' livelihoods and territory development. Environmental outcomes focus on sustainable farming and food miles, and social outcomes focus on social justice and political action [38]. [52] assess economic, social, and environmental sustainability factors in AFNs. Critical economic factors were improving the outlook for farmers' growth, starting relationships between consumers and producers, and intensifying the link to the local economy. Social factors that stood out included agritourism, inclusion, improved commitment, and improved information regarding nutritional value. A life cycle analysis study by Ref. [53] shows that AFNs have both benefits and challenges regarding sustainability, and the results suggest that optimisation techniques, process improvements and digital technology can play a role in the sustainable benefits of improving supply chains in AFNs.

### 2.3 Supply chain quality management practices in global food systems

Food SCQM requires traceability, trust, quality monitoring, and the adoption of emerging technologies [54,55]. Also important are the practices for food SCQM [56,57]. Practices in supply chain management research have been categorised. For example [58], identify six categories: supply chain integration, information sharing, customer service, customer relationships, supplier relationships, and postponement. Concerning food, the SCQM practice categories are supplier quality management, top management leadership and commitment, human resource management, quality of information and information system management, supply chain integration, customer focus, and internal quality management (i.e. process management and logistics management) [35,59]. Consumer quality perceptions drive SCQM practices in response to their needs (i.e. diets, religion, values) and desire for more sustainable, high-quality food supply chains [60]. Table 2 presents an overview of food SCQM practices in the literature associated with food supply chains in global food systems.

**Table 2:** SCQM practices in global Food systems.

SCQM Practice Category	Food SCQM Practices	Source
Supplier quality management	The purchase of materials, contracts, improving supplier standards and consistency, supplier communication, supplier engagement, Supplier relationships, raw product quality, collaboration, supplier process, collaboration with suppliers to achieve sustainability goals (iso14000),	[34,35]
Top management leadership and commitment.	Internal managerial motivation, performance excellence, size of company, coordination, readiness, alignment with company objectives, built on mission and vision, clear guidelines, decision making, factual approach to decision making, investment, involvement of people, management review, preparing mindset, recognise and reward quality, establish robust quality systems, system approach.	[34,35]
Human resource management	Employee development, knowledge, skills, the responsibility of employees to ensure quality processes, and personnel empowerment.	[34,35]
Quality of information and information system management	Data sharing, system reliability, improved processes on information exchange, improved communication, information availability, tracking, Information flow, information sharing, information transparency, the interaction of data and actors, interoperability, due diligence, technology performance, system integration, usability, supporting business and supply chain activities.	[35]
Supply chain integration	Information sharing, cooperation with governmental organisations, hybrid relationships, market relationships, vertical integration, supply chain relationships, disintermediation, joint decision making, mutually beneficial for supplier-buyer, relationship quality, synergies between actors, trust, transparency, supply chain visibility, partnerships, long-term relationships, cross-functional integration, reputation.	[35]
Customer focus	Consumer protection, sales process, consumer demand, consumer health and welfare, consumer knowledge, customer confidence, customer satisfaction, improved customer complaint handling, improved sales, increased consumer willingness to pay, loyalty, reliability, close communication, forecasting, feedback, convenience, customer service.	[34,35]
Process management	Defining the product design space, defining the process design space, defining a control strategy, process validation, process monitoring, traceability, production and management process, process quality standardisation, efficiency, flexibility, improved recalls, improving the flow of product, process approach, process documentation, provenance, resource management, standardisation, maintain backorders, recycle, re-use, use appropriate food technology, reduce food waste.	[34,35]
Logistics management	Good warehouse practices, internal quality management, logistics and circulation process, food logistics process, lowering costs of logistics, receiving and storage assignment, and inventory management.	[35]
Quality control	Auditing, the complexity of standards, authentication, commitment, customised quality checking, detection of defects, diagnosis, eliminating the need for multiple audits, hybrid governance, improved control, improved identification of errors, improved monitoring, quality risks management, quality validation, regulatory compliance, standards and abstract guidance schemes, statistical process control.	[34]
Continuous improvement	Defective product reduction, governance, minimising errors.	[34]

The SCQM practices have been associated with enhanced food SCQM performance [35,61] and sustainability performance in food supply chains [59,62]. Adopting digital technologies is becoming essential for SCQM practices and performance in food supply chains [63]. Digital technologies, for example, AI, Blockchain, Big Data, and IoT, can enhance the traceability, trust, integrity, and provenance of critical process steps in food supply chains [64]. Several vital areas for integrating technology and SCQM include establishing digital platforms for customers and suppliers and using digital supply chain technologies to support performance and processes [65]. Digital traceability and transparency systems can effectively support SCQM and quality assurances in the supply chains [66]. Such technologies are often developed for large-scale, influential stakeholders in food supply chains [67]. Work is now emerging on digital technologies for SCQM in the AFN supply chains [68].

## 3 Methodology

### 3.1 Overall research approach

**Fig. 1:**
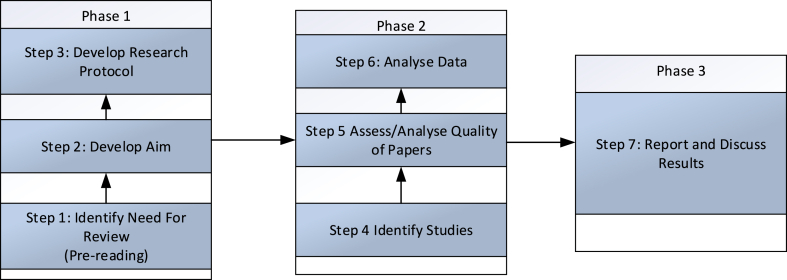
Slr phases and steps.

A systematic literature review is adopted in this work and aims to identify the practices for SCQM in AFNs. A systematic literature review is a systematic, explicit, reproducible method to identify, evaluate and interpret current publications and documents [69,70]. There are some important considerations before undertaking a systematic literature review, including the availability of literature in a domain, the absence of recent or high-quality reviews, and gaps in existing reviews [71], all of which are key considerations in the current review. In a systematic literature review, an integrative approach summarises existing literature and identifies patterns and topics concerning past publications. The main steps in the systematic literature review are defining the research problem and question, determining the characteristics of primary studies, retrieving potentially relevant literature, selecting pertinent literature, synthesising literature, and reporting results [69,72,73]. See Fig. 1 for the steps taken in this research.

### 3.2 Planning and protocol development

Prior to undertaking the systematic literature review, a research protocol for the review was developed with the research team. The protocol adapted elements of the PRISMA checklist to prepare and align the research. The title, rationale, research objectives, research questions, eligibility criteria (inclusion and exclusion criteria), information sources, search strategy, data management, section process, data collection process, outcomes, and prioritisation (including analysis type) were developed in this process. Phase one involved establishing the need for research, planning, and protocol development. Standardised search strings were applied, reviewed, and amended to find relevant papers for answering the research aim and questions. The search string that was adopted is TITLE-ABS-KEY ("Food" AND "Supply Chain" AND "Quality" AND "Management"). The reference manager, Mendeley, was used. Science Direct, Scopus, and Emerald Insight are the three chosen databases for this study. Scopus can be referred to as a justified database as one of the two most extensive databases of scientific articles. Reviewers using systematic literature review methods are recommended to use two or more databases. Based on a search most recently updated in early January 2023, 1873 hits for further filtering and analysis were identified. Selected studies for full paper review were put in a combined PDF document for review and analysis.

### 3.3 Selection of papers and quality assessment

According to Ref. [73], determining the required characteristics of a primary study is an essential step in systematic reviews. The basis of inclusion and criteria should align with the primary objective of the research. This review identifies SCQM practices in AFNs and examines their correlation to AFN supply chain quality conventions. Therefore, this research applies inclusion and exclusion criteria tailored to achieve this aim. Given the narrow scope of the paper (focusing on supply chains in AFNs), the criteria for the unit of analysis, the method employed in the selected papers, and the quality of journals are widely established. The keywords and Boolean operators were established by studying relevant and related literature review studies. Other areas to consider in setting up the review process include search periods, search fields, subject area, document type, language, document relevance, and the selection of other inclusion and exclusion criteria to support the aim [74].

**Fig. 2:**
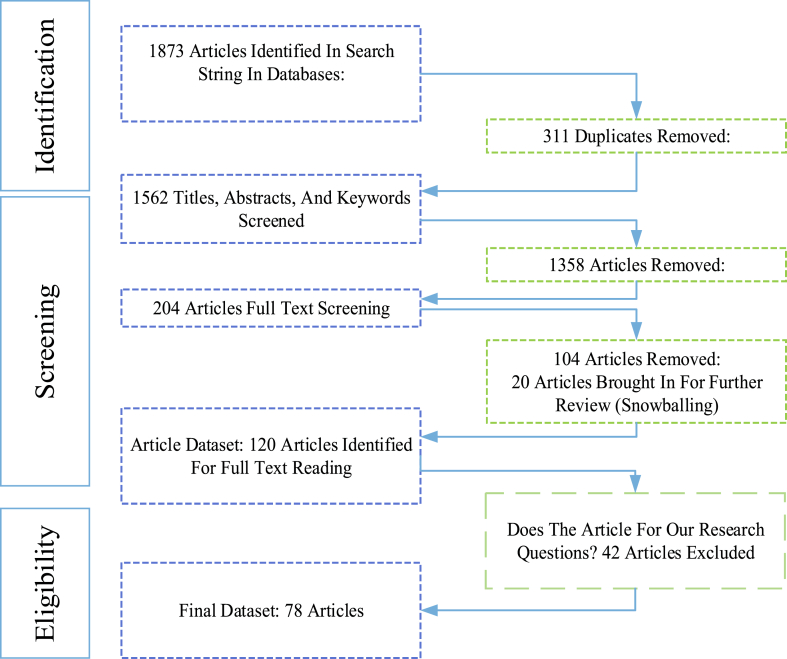
Search results and paper selection process.

Before the screening process, the researchers evaluated and agreed upon inclusion and exclusion criteria. The basis for inclusion criteria included the following features. Papers were selected based on a 20-year time frame from 2003 to 2023. Only peer-reviewed articles were used in this paper. Grey literature was excluded. Mainly, journal papers were included, with the allowance of a few highly relevant conference proceeding papers. Finally, keywords were set in the protocol phase and used to screen out irrelevant documents. The specific inclusion and exclusion criteria are shown in Table 3, and the related keywords used in the screening process are in Table 4. The inclusion and exclusion criteria were followed strictly to meet the quality assessment goal. Quality assessments should a) explore subjective meanings relating to the experience of others, b) select papers systematically, c) provide understanding and interpretation of data, and d) findings made within papers should be supported by the data presented. During the final screening process, 20 additional papers were identified for inclusion in the review. This was done by applying the snowballing approach. Fig. 2 shows the search results and screening results for this research.

**Table 3:** Inclusion and exclusion criteria.

Inclusion criteria: General Criteria: English documents only. Peer-Reviewed. Shows contribution. Not before 2003. 1st Screen: Title: Abstract: Keyword Criteria: The Title, Abstract, or Keywords contain Alternative food Networks (Or Related Key Words) 2nd Screen: Title: Abstract: Keyword Criteria: Does the article contain information about at least two key research terms (Or Related Keywords), i.e. Food Supply Chain Quality Management AND Alternative Food Networks? 3rd Screen: Full-Text Review: Does the article fit the scope of the research by offering insight into supply chain quality management practices in alternative food networks?
Exclusion criteria: Non-English Language. Only peer-reviewed papers and no grey literature Older than 2003. It needs to fit the scope of research by providing more insight into supply chain quality management practices in alternative food networks.

**Table 4:** Keywords used in the screening process.

“Quality Management” OR	“Quality” OR “Food Quality” OR “Quality Assurance” OR “Quality Control” OR “Quality Improvement” OR “Quality Assessment”	[75] ′
“Alternative Food Network” OR	"Community-supported Agriculture" OR "Farmer Markets" OR "Organic Food" OR "Cooperatives" OR "Farm Shop" OR "City-Region Food" OR "Urban Agriculture" OR "Box Schemes "OR Community Gardens" OR "Short Food Supply Chain" OR "Local Food" OR "Food (Re) localisation" OR "E-Commerce" OR "E-Supply Chain"	[17,42]

### 3.4 Analysis and reporting results

Data was extracted using a standardised format, including research focus, link to SCQM practices, link to sustainability, and link to the quality conventions of AFNs. The extracted data was synthesised using inductive content analysis to bring forth the SCQM practices in AFNs. The data like year of publication, journal name, and author(s) were also collected. The approach draws from existing templates to ensure the results are consistently drawn forth. Such an approach can contribute to robustly understanding the material and guide the research process from a scanning stage to a more substantial stage of analysis [76]. The analysis of the results is in three parts. First, descriptive statistics show i) publication by year, ii) category of AFNs covered in the papers, and iii) top journals by the number of papers included. Second, a content analysis was adopted and used to categorise SCQM practices identified in related work. The content analysis used to code using the main food SCQM practices categories identified in related work [34,35]. These are supplier quality management, top management leadership and commitment, human resource management, quality of information and information system management, supply chain integration, customer focus, internal quality management, and quality control and governance. Practices found within the included sample linking to those categories were identified and placed within each practice category. The third part was to report on and discuss emerging themes.

## 4 Results

### 4.1 Descriptive statistics

**Fig. 3:**
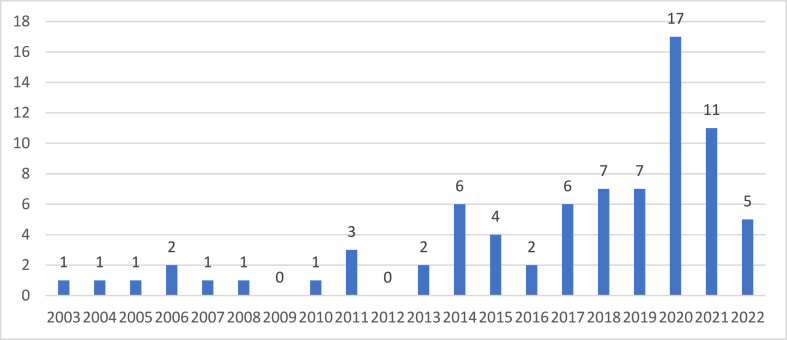
Journal Publication by year.

**Fig. 4:**
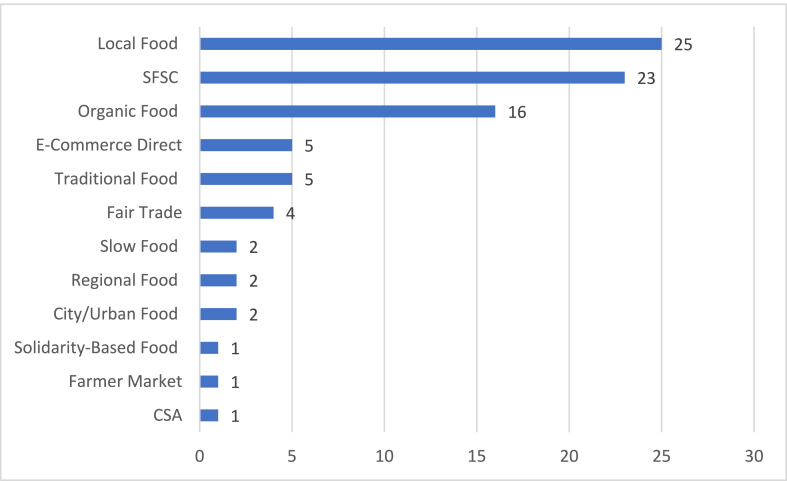
Publications by categories of AFNs.

Fig. 3 shows the Journal Publications by Year. The keywords linked to categories of AFNs represented within the papers are presented in Fig. 4. Local food, followed by short food supply chains, and organic food were the frequently mentioned categories throughout the papers. Some of the papers focused on more than one AFN category.

The 78 articles were spread across 48 journals, and three conference papers related strongly to the current research. The top five journals where papers were used in the analysis phase are Sustainability, British Food Journal, Supply Chain Management, Journal of Rural Studies, and Food Quality and Preference, see Table 5.

**Table 5:** Number of publications by journal.

Journal	Number of Publications Cited
Sustainability	13
British Food Journal	5
Supply Chain Management	4
Journal of Rural Studies	3
Food Quality and Preference	3
Environment and Planning A	2
International Journal of Information Management	2
International Journal of Hospitality Management	2
International Food and Agribusiness Management Review	2
*Other	42

*IIE Annual Conference. Proceedings; Global Food Security; Renewable Agriculture and Food Systems; Journal of Environmental Studies and Sciences; International Journal of Integrated Supply Management; Foods; International Journal of Supply Chain Management; Journal of Food Products Marketing; IEEE Conference; Land Use Policy; European Countryside; Concurrency Computation Practice and Experience; Discrete Dynamics in Nature and Society; International Journal on Food System Dynamics; Animals; BMC Public Health; International Journal of Production Economics; World Review of Entrepreneurship, Management and Sust. Development; Agronomy for sustainable development; Appetite; Advance Journal of Food Science and Technology; International Journal of Production Research; Agricultural Systems; Agriculture and Human Values; Agronomy; Journal of Cultural Economy; Revue de Geographie Alpine; Service Industries Journal; Geografiska Annaler, Series B: Human Geography; IOP Conference; Toxicology; IEEE International Conference on Universal Village; Chemical Engineering Transactions; Scientia Horticulturae; Journal of Enterprise Information Management; Journal of Destination Marketing & Management; Journal of Cleaner Production; Socio-Economic Planning Sciences; Procedia Computer Science, Sustainable Production and Consumption, Sustainable Cities and Society, Total Quality Management and Business Excellence; Sociologia Ruralis*.*

### 4.2 Summary of papers

**Fig. 5:**
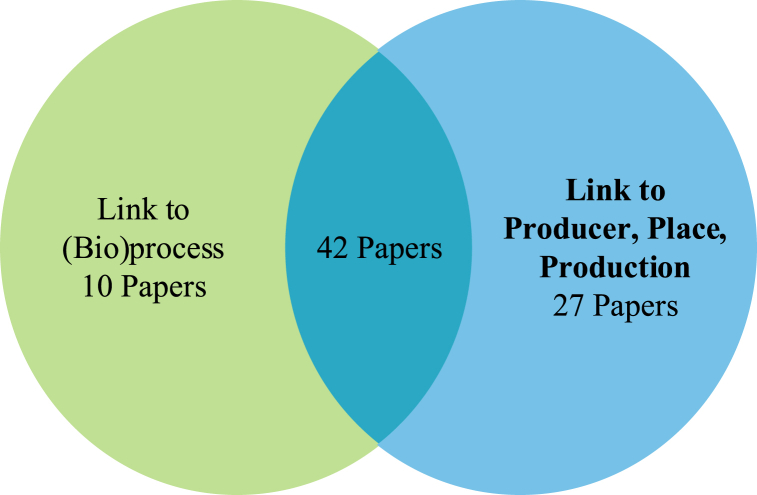
Overview of papers linking to the quality conventions in AFN

Table 6 summarises the focus of the selected papers, the link to quality and sustainability, and the AFN quality conventions. The papers link to the quality conventions (bio)processes and producer, place, and production. Fig. 5 shows these links.

**Table 6:** Overview of publications.

Source	Stakeholder perspective	Research Focus	Link to SCQM practices	Link to Sustainability	Link to Bioprocess	Link to Producer, Place, Production
[77]	Consumers	Consumer preferences	Product and process quality	Not Specified	X	X
[78]	Company	Digitalisation	Supply chain relationship and integration quality	Not Specified		X
[79]	Company	Success factors	Quality of product and processes.	Not Specified		X
[80]	Company	Framework for supply chain quality	Logistics quality	Not Specified	X	X
[81]	Public (School)	Criteria for food procurements	Product and process quality	Social, Environmental, Economic	X	X
[82]	Company	Inventory management and shelf life	Enhanced quality through fresh produce inventory optimisation	Economic		
[83]	Consumer	Sustainable local food systems	Quality perception (health, taste, freshness)	Social, Environmental, Economic	X	X
[84]	Company	Supplier selection in local food systems	Supplier quality and supply chain relationships	Not Specified		X
[85]	Company	Quality management in organic supply chains	Integration and quality control	Not Specified	X	X
[86]	Multi-Actor	Local embeddedness of producers and consumers	Flexible quality governance schemes	Social		X
[87]	Company	Relationships between supply chain actors in an organic supply chain	Food quality and traceability	Not Specified	X	X
[88]	Multi-actor	Climate resilience through new food systems	Product Quality	Social and environmental	X	X
[89]	Company	Cultivation and production in AFN	Production quality	Not Specified		
[90]	Multi-Actor	Overview of short food supply chain	Product quality, fresh, safe	Social, Environmental, Economic	X	X
[91]	Consumer	Product quality in local food supply chains	Intrinsic product quality	Not Specified	X	X
[92]	Company	Structures of Organic Commodity Supply Chain in Europe	Risks in quality safety, trust, and supply chain relationships	Social, Environmental, Economic	X	X
[93]	Company	Innovative techno-managerial activities in AFN	Quality requirements of stakeholders	Not Specified		X
[94]	Company	The organisational arrangement in AFN	Direct relationship, geographical indication (PDO), direct marketing, quality food, information exchange, transparency, communication, trust	Social		X
[95]	Company	Use of centralised logistics for local producers	Logistics, product quality, geographical indications, quality production			X
[96]	Multi-Actor	SFSCs concerning the triple bottom line	Geographical indications (PDO, PGI), fair, trust.	Social, Environmental, Economic	X	X
[97]	Company	Potentials and constraints of an alternative food networks	Quality, taste, appearance, ethics, variety, good farming practices	Not Specified	X	X
[98]	Multi-Actor	Blockchain in local food	Product quality, traceability, provenance, transparency, trust	Not Specified		X
[99]	Company	Blockchain-based e-commerce supply	Product quality and safety, traceability, relationship quality, resource integration	Economic		X
[100]	Multi-Actor	The sustainability of a food system	Geographical indication and origin labelling	Social, Environmental, Economic		X
[101]	Multi-Actor	Performance of value chains of dairy farms,	Geographical indications and origin labelling; certifications quality and production quality		X	X
[102]	Company	Quality in organic food supply chains	Product quality, quality standards, process, distribution, retailing, cultural, ethical, and supplier quality.	Social, Environmental, Economic	X	X
[103]	Multi-Actor	Local food sourcing for public schools.	Product Quality	Not Specified	X	
[104]	Company	Governance of geographical indications and supply chain ownership and their effect on quality performance	Geographical indications (PDO, PGI), collaboration	Not Specified		X
[105]	Company	Digitalisation and geographical indication	Quality of supply chain processes, geographical indications (PGI, PDO), collaboration, and integration.	Economic		X
[106]	Company	Harvesting practices impact food quality	Geographical indications (PDO, PGI), product quality, supplier quality, packaging quality, quality requirements, quality control goals, and production/harvesting quality management.	Not Specified	X	X

[107]	Multi-Actor	Relationship quality and supply chain performance	Relationship quality, geographical indications and product origin, product quality and safety	Economic	X	X
[108]	Company	Quality-related decisions in and amongst supply chain stakeholders	Transparency, trust, regulatory quality, supplier quality, freshness, quality control, protection quality	Not Specified		
[109]	Company	Supply chain quality integration to enable organisational ambidexterity	Internal quality integration, supplier quality integration, customer quality integration, relationship, and employee involvement;	Not Specified	X	X
[110]	Company	Traceability technology for food supply chains	Traceability, geographical indication, consumer-driven,	Economic	X	X
[111]	Not specified	Territorial production of foodstuffs	Geographical indications	Not Specified		X
[112]	Consumers	Perceived quality in traditional food supply chains	Quality production standards, traceability systems, quality certification, personal relationships, country of origin	Social	X	X
[113]	Not specified	Food safety and quality microbiological hazards in the SFSC	Food safety, food quality;	Not Specified	X	
[114]	Company	Alignment of product and supply chains	Product Quality	Not Specified		
[115]	Company	Vegetable cropping systems in the agri-food supply chain.	Quality product, production quality	Not Specified	X	X
[116]	Company	Ethical poultry production in the food supply chain.	Quality control, quality conventions (price, promotion), ethics', quality products, animal welfare	Social and environmental	X	X
[117]	Multi-actor	Development of a local sales system based on the SFSCs	Production quality, supplier quality, supply quality, supply availability, transparency, relationships, trust, loyalty, quality guarantee, communication, cooperation, interpersonal relations	Social	X	X
[118]	Company	Compare organic and non-organic production practices in food supply chains	Communication, cooperation, interpersonal relations, product quality	Social, Environmental, Economic	X	
[119]	Company	*Trans*-local quality standards in the coffee supply chain,	Certification quality, fair trade, quality conventions, quality control, process quality, production quality,	Not Specified	X	X
[120]	Multi-actor	Socio-historical analysis of the institutional context for developing the value chain context in a specific traditional area	Geographical origin, geographical indication, original, quality production, quality control, quality of product, quality valorisation, quality differentiation, territorial quality, trust;	Social	X	X
[121]	Company	Private label products and governance	Geographical indications (PDO, PGI), supply chain governance, contracts, standards, private labelling, trust, food quality, food safety	Economic		X
[122]	Company	Attributes for selecting fresh fruit and vegetable suppliers for retailers	Buyer-supplier relationship, direct trade relationships, supplier section criteria, goodwill, certification, geographical location, long-term relationships, brand name, product quality, product consistency, product variety, supply availability, packaging, organic, food safety, traceability, distribution quality		X	X
[123]	Company	Traditional and supermarket-driven value chains	Product quality, standards, reliability, GLOBALGAP, seasonality availability, quality control, supply chain relationships, quality inspections, GMP	Social	X	X
[124]	Not specified	Blockchain technology to enhance traceability	Quality costs, traceability, supply chain cooperation, trust, improved information	Economic		X
[125]	Not specified	Regulations set for food quality and safety	Food safety, food quality, quality management system, regulations, transparency,		X	
[126]	Company	Bio-markers to provide a solution to the digital-physical boundary	Visibility, biomarker, fair trade, organic, data quality, product quality, origin, material quality, trust	Environmental	X	
[127]	Multi-actor	Supply chain structures and technology for agri-food supply chains	Product Quality	Not Specified		
[128]	Multi-actor	For data-driven urban agriculture	Product quality through freshness and variety	Not Specified	X	
[129]	Certifiers	The role of standards and certification in alternative food networks	Standards and certification, governance quality schemes guarantee “process” quality abstract labelling schemes, certifications	Economic	X	X
[130]	Company	Assess supply chain performance of electronic-based agricultural supply chains.	Product quality, reliability, supply chain relationships	Not Specified		X
[131]	Multi-actor	Business models in AFNs for enhanced sustainability	Supply chain relationships	Social, Environmental, Economic		X
[132]	Company	Critical Success Factors in SFSCs	Geographical identifications, specificity of territorial brands, direct buyer-supplier relationships, organic production, food safety, traceability, cultural heritage, consumer health, product origin, local work, cooperation,	Social, Environmental, Economic	X	X

[133]	Not specified	Differences between SFSCs and their role as local, sustainable systems.	Quality, labelling, valorisation, value, health, fresh, taste, values	Social, Environmental, Economic	X	X
[134]	Multi-actor	Sustainability of global food supply chains vs. local food systems.	Food quality and safety management systems (ISO, HACCP, GLOBALGAP BRC), geographical identifications (PDO, PGI), certification, personal relationships, private labels, environmental footprints, large cooperatives, green labels, and nutrition metrics.	Social, Environmental, Economic	X	X
[135]	Consumers	Value of local food from a tourist's perspective	Supplier knowledge, supply chain interaction, quality products	Social	X	X
[136]	Consumers	The willingness to pay for local products	Price/quality, locally sourced, perceived quality, freshness;	Economic	X	
[137]	Multi-actor	Trust and personal relationships between stakeholders	Trust, personal relationships, quality products, rapport quality	Social		X
[138]	Multi-actor	The quality conventions at local food markets in the UK	Trust, supply chain relationships, personalised relationships, local production, regard convention, interpersonal			X
[139]	Company	Boundary conditions for blockchain adoption e-food supply chain	Traceability, food quality, food safety	Not Specified	X	X
[140]	Company	Blockchain in delivery and distribution management	Traceability, information sharing, trust, transparency, immutable, use of smart contracts, product quality	Not Specified	X	X
[141]	Company	Prototype model of blockchain-based traceability to ensure food quality in restaurant	Product quality, product safety, the origin of food, quality control, standards, traceability, quality grading		X	X
[142]	Not specified	Blockchain technology in the food industry	Quality control, quality guarantee, traceability, transparency.			X
[143]	Consumers	Constructs of quality in SFSCs	Trust, knowledge, direct interaction, local origin, geographical identifications, certification;	Social		X
[144]	Not specified	Trends for local food supply chain	product quality, process quality, freshness	Not Specified		X
[145]	Governing bodies	Certifications in local food economies	Geographical indication and designations (PDO), quality certifications, provenance, product quality, quality guarantee, quality assurance, organoleptic quality	Economic	X	X
[146]	Company	Relationship quality in national organic food supply chains.	Vertical coordination, relationship quality, trust, horizontal coordination, organic food, product quality	Economic	X	X
[147]	Consumers	Consumer preference for local and types of quality perceptions	Knowledge over producer, place, and production practices, process attributes, shipping, animal welfare, feed types, product attributes, food safety, freshness, nutrition, social goods, origin, trust, and personal/direct relationships;	Social, Environmental, Economic	X	X
[148]	Company	Blockchain in the biodynamic food supply chain	Trust, visibility, traceability, governance	Not Specified	X	X
[149]	Company	Geographical identification of SFSCs Italy	Geographical indication (PDO), training, traceability, trust	Social	X	X
[68]	Multi-actor	Blockchain-enabled Enabled Quality Management Architecture in SFSCs	Promote fairness, ethical and just practices, improve health and safety, prove specified geographical area and authenticity, manage product and process quality, enhance freshness, support trust, transparency and relationships, use of digital technologies.	Social Environmental	x	x
[36]	Multi-actor	Co-production cooperatives in AFNs;	Commitment, coordination, connection with farmers, knowledge, and trust, information technology, involvement, participatory guarantee systems, participation in decision-making,	Social, Environmental	x	x
[150]	Consumers	Pre and post covid consumer value perception in SFSCS	food safety, freshness, and enhanced product quality.	Social, Environmental	x	x
[151]	Not specified	A proposed IoT system to help understand traceability in the food supply chain.	Track and trace, Information technology			x
[152]	Company	Chain management mechanisms in German wine supply chains	Contracting, incentives, ownership, monitoring and sanctions, collaboration, embeddedness communication, trust, collective strategy, social capital, power, reduce anonymity, relationships, leadership, programming, commitment, culture, IT, Dedicated alliances, Feedback, training and development, teams and groups,	Social		x

### 4.3 Supply chain quality management practices

**Fig. 6:**
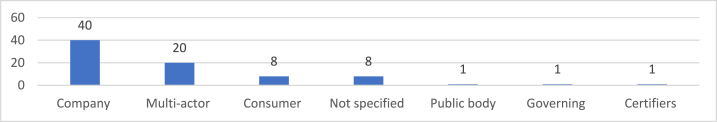
Stakeholders perspective across papers.

Practices were found to arise from different areas of the supply chains, with most studies focusing on companies, multi-actors, and consumers. See Fig. 6.

The SCQM practices found in the literature for AFNs are in Table 7, including the number of papers that mention the practices. The most identifiable practices include the geographical indication of production/provenance under the supplier quality management category, followed by the quality of raw materials. Adopting digital technologies and information sharing/flow are highly identifiable in the reviewed papers for the quality of information and information system management category. Most papers mention trust between stakeholders for the supply chain integration category, followed by supply chain relationships, direct relationships, and cooperation/collaboration. Transparency is also highly identifiable, with various papers focusing on this practice. Product quality is frequently mentioned under the customer focus category throughout the included papers, while product safety and communication with customers are also important. Internal quality management focuses on traceability, production quality, and process quality. The quality control category shows quality schemes related to geographical origin (PDO/PGI) and quality management systems (i.e. ISO, GLOBALGAP, IFS, BRC, HACCP. QGAP) in a considerable number of papers. Also identifiable in multiple papers are quality control, governance, and auditing.

**Table 7:** SCQM practices for alternative Food networks*.

Main SCQM Practice Categories	Sub-Categories of SCQM Practice AFN (Frequency of occurrence in related papers)	Source(s)
Supplier Quality Management Practices	Geographical Indication (Origin) Of Production/Provenance (28); Raw Material Input Quality (5); Supplier Knowledge (4); Reputation Management (3); Locally Grown Produce (3); Supply Availability (2); Supplier Reliability (2); Supplier Price/Quality (1); Quality Producers (1); Good Farming Practices (1); Supplier Quality Integration (1); Supplier Creditability (1); Supplier Consistency (1)	[[36], [77], [79], [83], [84], [91], [94], [95], [96], [100], [102], [104], [105], [106], [107], [108], [109], [110], [111], [112], [117], [120], [121], [123], [126], [132], [133], [136], [138], [141], [143], [145], [147], [149]]
Top Management Leadership and Commitment Practices	Commitment (4); Quality Management Strategy (3); Integrated Management (1); De-centralised Quality Decisions (1)	[36,85,105,107,108]
Human Resource Management Practices	Training (2); Employee Involvement (1)	[109,149,152]
Quality of Information and Information System Management Practices	Adoption Of Enabling Technologies (16); Information Sharing/Flow (8); E Supply Chain (3); Smart Contracts (2); Architecture (3); Data Quality (2); Trust-Worthy Systems (2); Information Security (2); Automation (2); Tamperproof (2); Traceability System (2); Digital Twin (2); Data Driven (2); Transparent Data (2); Immutable (2)	[[36], [68], [80], [87], [94], [98], [99], [107], [110], [124], [126], [127], [128], [130], [134], [140], [141], [148], [149], [151]]
Supply Chain Integration Practices	Trust Between Stakeholders (21); Supply Chain Relationships (13); Direct Relationship (10); Cooperation/Collaboration (9); Transparency (9); Buyer-Supplier Relationships (3); Integration (3); Vertical Relationships (2); Quality Of Relationships (2); Traditional (2); Visibility (2); Coordination (2); Participation (2); Contracts (2); Stakeholder Embeddedness (2); Social Agriculture (1); Social Embeddedness (1); Communication (1); Supply Chain Ownership (2); Consumer Involvement. (1); Loyalty Between Stakeholders (1); Dispute Resolution (1). Promote Fairness (1); Ethical and Just Processes (1); Dedicated Alliances (1)	[[36], [68], [78], [79], [83], [84], [85], [86], [87], [91], [92], [94], [96], [98], [99], [105], [107], [112], [117], [120], [121], [123], [126], [131], [132], [134], [137], [138], [143], [146], [152]]
Customer Focus Practices	Food Quality (Intrinsic and Extrinsic Attributes) (32); Food Safety (13); Communication with Customers (11); Consumer Driven (4); Variety (4); Price/Quality (3); Guarantees (3); Quality Differentiation (2); Consumer Preferences (1); Authentic Product and Process (2); Provide Fresh Product (2); Consumer Demand (1); Direct Marketing. (1); Customer Quality Integration (1); Quality Conventions (1); Original Product Offerings (1)	[[68], [77], [79], [83], [88], [89], [90], [91], [95], [96], [97], [98], [102], [103], [108], [112], [113], [114], [120], [121], [122], [125], [127], [128], [129], [132], [133], [134], [135], [136], [138], [139], [141], [144], [149], [150]]
Internal Quality Management Practices	Traceability (14); Production Quality (9); Process Quality (7); Logistics Quality (4); Packaging Quality (4); Production Standards (3); Inventory Management (1); Shelf-Life (1); Flexibility (1); Resource Integration (1); Compliance (1); Distribution Quality (1); Retailing Quality (1); Responsiveness (1); Internal Integration (1); Warehousing Quality and Control (1); Standardisation (1)	[68,80,87,89,98,99,101,108,110,112,115,119,122,132,139,141,142,144,151]
Continuous Improvement Practices	Zero Product Defects (1)	[134]
Quality Control and Governance Practices	Quality Schemes Related to Origin (PDO/PGI) (13); Quality Systems and Standards (Iso, GlobalGap, IFS, BRC, HACCP. QGAP) (9); Auditing and Inspections (5); Participatory Governance Approach (6); Quality Valorisation (3); Quality Control Methods (2); Quality Protection (2); Statistical Tools (1); Flexible Quality Governance (1); Quality Requirements (1); Quality Control Goals (1); Good Manufacturing Practices (1) Science-Based Measures (1); Biomarker (1); Quality Grading (1); Monitoring and Sanctions (1)	[[68], [77], [84], [85], [93], [94], [96], [100], [102], [104], [105], [106], [112], [116], [118], [119], [120], [121], [123], [125], [129], [134], [145], [149], [152]]

## 5 Emerging themes

**Fig. 7:**
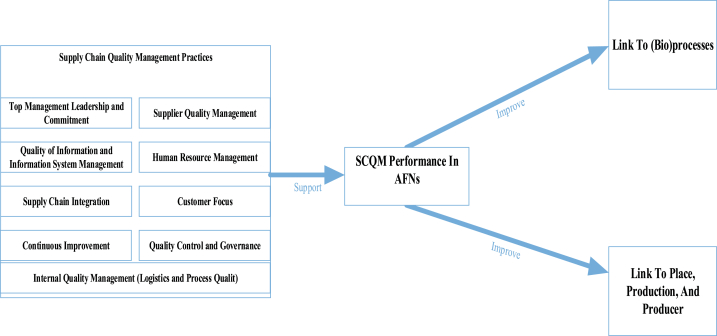
Framework for supply chain quality management In AFNs.

Based on the results in Tables 7, it becomes apparent that although the SCQM practice constructs from global food systems are helpful in SCQM in AFNs, the practices have differences, reflecting the need for transparency, close relationships, trust, and geographical indications of products. Based on the literature, there is some evidence that SCQM practices can support SCQM performance in AFNs, as shown in the framework illustrated in Fig. 7. The emerging themes are based on the relationship between SCQM practices, SCQM performance, and the link to quality conventions of AFNs, as shown in Fig. 7.

### 5.1 Practice-based Evolution of supply chain quality management and performance

Supply chain quality management offers an ability to enhance quality performance in AFNs [109] through practices like a geographical indication of origin/provenance and governance schemes [104], supply chain collaboration and relationships [85,92], traceability [87], and adoption of emerging technology, i.e. blockchain [99]. Provenance includes information and the understanding of the geographical origin of a product, in addition to demonstratable transparency from the producer to the end consumer [153], thus providing better information for consumers over suppliers and their reputation. Geographical indications in food supply chains play an essential role in the governance of SCQM in AFNs and lead to higher quality performance levels [104]. Quality governance in AFNs can be supported through institutionalised schemes like PDO and PGI that set standards for processes to support higher-quality performance levels. Relationship-based governance and trust are also important for quality in AFNs [105], which may be supported by using flexible and participatory based practices [86,104], and the adoption of participatory guarantee systems [154]. Governance may also be enabled through supply chain integration, for example, vertical integration and moving processes backwards to suppliers or forwards towards customers [121], removing the need for intermediates. Strong relationships are required in vertical integration and its governance structure. Supply chain integration and relationships contribute to SCQM and performance in AFNs [92], for example, by supporting governance, quality integration, and ambidexterity [107,109,121]. Some barriers to supply chain integration are a lack of trust, unaligned goals between stakeholders, and loss of control [121]. Trust, personal buyer-supplier relationships, traceability, and transparency are essential to reinforce supply chain integration and relationships [87,96]. Human resources, continuous improvement, and top management and leadership quality were rarely shown throughout the literature as practices for SCQM in AFNs, but they may play a supporting role.

### 5.2 Relating SCQM practices to quality conventions in AFNs

**Fig. 8:**
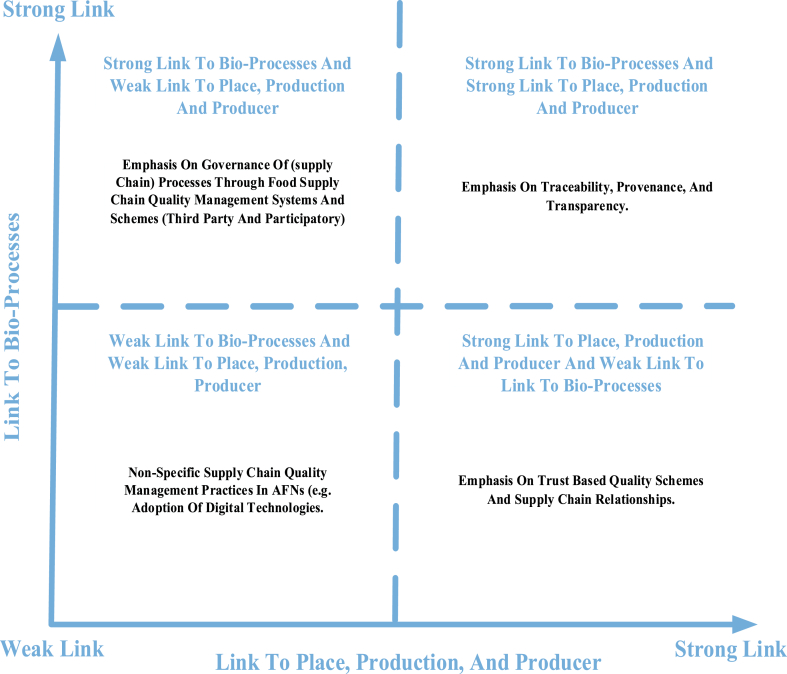
Framework for linking SCQM practices to quality conventions and schemes in AFNs.

Based on the link to producer, place, production, and link to bio (processes), a framework for linking these quality conventions of AFNs and SCQM practices is proposed. See Fig. 8. Four quadrants are shown. The top-right quadrant is a strong link to (bio)processes and a strong link to place, production, and producer. In this quadrant, SCQM practices should emphasise traceability, governance, provenance, and transparency to control the quality of products and processes, from producer to consumer. Practices that are built around trust and supply chain relationships are in the bottom right quadrant. Supply chain relationships and trust can be strengthened through direct links between producers and consumers or disintermediation. As AFNs develop to more extended supply chain types, the adoption of emerging technology may support this. The top left quadrant represents the practices linking to (bio)processes, showing the importance of food SCQM systems and schemes. These systems might be based on structural assurances (e.g. PDO, PGI, ISO, BRC), or participatory guarantee systems. The bottom left quadrant represents a weak link to the AFN quality conventions. Although there is little to link these SCQM practices to the place, production, producer, or (bio)process, these practices may be required or can enable SCQM in AFNs. An example is the adoption of digital technologies, which could enhance traceability, governance, and support trust. The emerging framework shows promise to assess the relevance of SCQM practices in AFNs through a stakeholder perspective by ranking the relationship between SCQM practice and quality conventions, which is a consideration for future work.

### 5.3 Improving the links in the quality conventions

The SCQM practices identified can reinforce the link to place, production, and producer. They reflect on the standards, norms, and values of the AFNs [42]. Provenance and geographical identification of production are essential SCQM practices to support links between consumers and upstream supply chain members (i.e. farmers) and create differentiation between chains [77]. Geographical indications and associated processes impact the quality of an end product [104], providing clear information about the producer and production practices [147]. Using geographical indication schemes can also increase the profits for producers [145], thus leading to a more fair price/value for upstream supply chain stakeholders. Setting specific outcome objectives and quality assessment criteria, adopting resource objectives towards a stable quality throughput and building the quality and coordination between actors [106].

In some AFNs, like the short food supply chain, consumer understanding of the place of production is a critical success factor [79,132]. Clustering/aggregating products between stakeholders is also possible as long as information on geographical aspects of production is not distorted [95]. This aggregation may lead to higher performance levels from enhanced logistic processes. In addition, quality schemes and labels (i.e. PDO, PGI) can communicate the locality of a product to the consumer and ensure processes are upheld. Supporting the work by Ref. [155] stating that consumers may need help understanding the difference between what is local and what is locality. Locality considers the geographical limits of inputs and production processes, where products can be sold at national and international levels [134]. Quality schemes often support the locality of a product [156,157]. Trust and direct relationships may facilitate local stakeholders more than standards and systems [29]. They may benefit from governance, assurances, and controls customised for SCQM in more localised initiatives in AFNs.

Trust is a complex notion and is an essential element of quality in AFNs, and it can reinforce consumer behaviour and confidence in food quality and safety [85,158]. Trust is a prerequisite for collaboration between stakeholders [92] and can contribute to the overall performance of the AFN [107]. Trust between buyers and suppliers is essential for quality management performance within food supply chains, as the erosion of trust occurs when there is a negative perception of SCQM [159]. [12] define trust concerning quality in AFNs through three key concepts: credibility, integrity, and benevolence. The researchers show that the more intense the supply chain relationship, the more focus is on benevolence, while less direct relationships lead to a need for integrity and accessibility. There is limited face-to-face interaction between the food producer and end consumer in the supply chains of global food systems. Therefore, abstract guidance schemes and institutional set quality standards support trust [160]. Within AFNs, trust in quality is more developed through personalised relationships, embedded information, and direct producer-customer interaction [161,162].

There is a link between trust, supply chain relationships, and quality management in food supply chains [57,163], with the structure and development of supply chain relationships taking a role in SCQM, particularly concerning contract stipulation [164]. In AFNs, the need for formalised contracts is sometimes replaced through trust building and long-term relationships between buyers and suppliers [87]. Relationships in the AFN are based on fairness, direct interaction, and supply chain integration [87,165]. Nonetheless, contracts can benefit AFNs [85] and act as a mechanism to structure transactions between supply chain stakeholders [166]. In the AFN, fairness between supply chain stakeholders is an essential consideration in building relationships [167], as a collaborative approach can be used to improve bargaining power [161], and this should be supported in formal contract agreements. Transparency enables trust in many AFNs with face-to-face interaction and direct relations [138]. However, as AFNs progress, a need for improved transparency is increasing, leading to a need for upgraded quality management through emerging digital technologies. Trust and supply chain relationships also contribute to consumer-driven product quality. Product quality in AFNs is associated with a perception of quality through taste, health attributes, and freshness [79,83,135]. Many of these elements link back to relationships and trust between buyers and suppliers, thus developing a trend for consumer-driven quality.

Traceability is a key concept in the quality management of food [168] and is an essential practice for SCQM in AFNs [87,112] by reinforcing consumer confidence in quality and trust [66]. The need for traceability in food supply chains is well recognised in the existing literature to support geographical indication [157], improve end-to-end monitoring of the supply chain [169], give confidence to the consumer [160], to help quality management systems and governance [170], and improve food safety [171]. AFNs enable traceability through more direct linkages and shorter supply chain constructs [96]. Consumers in AFNs demand improved levels of traceability [26] to ensure quality aspects in AFNs, for example, ethnicity, authenticity, ethnicity, and locality/localness of raw materials [24,132], proof of processes, origin and quality certifications, thus coming back to traceability needs [145].

SCQM practices can support a link to bioprocess (i.e. transparency, governance, standards, auditing and controls, product quality management, product safety, and traceability). Some practices, e.g. governance, extend beyond the link to place, production, producer, and link to (bio)processes. A need for governance and quality controls is important for the link to (bio)processes [104], considering the process and product quality and standards set in the AFN [102,143]. National and subnational quality standards have been set to support and control the product, process, and product quality in AFNs, i.e. SALSA (Safe and Local Supplier Approval) has been introduced in the United Kingdom to support SMEs that generally have difficulties meeting traditional quality management systems like BRC and IFS [29].

Participatory guarantee systems are also emerging and offer an alternative to institutionalised quality management systems [24]. The participatory guarantee system reflects on the peer-to-peer, knowledge-based governance systems defining quality practices at local levels, set outside of formally constructed structures [154]. A participatory guaranteed system offers an opportunity to act as an innovative governance structure for AFNs, with the potential of replacing institutional set systems. Important in participatory guarantee systems is that standards are clear and strict for governance and clearly define sanctions for non-compliance [172]. Participatory guarantee systems support the need for flexible quality governance between initiatives [86]. [129] examine the governance of face-to-face, proximate, and extended AFNs, suggesting that quality schemes provide guarantees for products and the qualities that those products have.

The quality management practices above linking to place, production, people, and (bio)processes can be enhanced through emerging techniques (bio-markers) and digital technologies (IoT, blockchain) [126,141]. For example, to enhance levels of transparency, information flow, immutability and traceability [99,124]. Blockchain can support practices like supply chain integration, transparency, traceability, supply chain relationships, and performance through the application, i.e. smart contracts, visibility, and non-repudiation [98,148]. Blockchain characteristics such as disintermediation, tamper-proof, trust-less, smart contracts, reliable and transparent information flow, immutable, and non-reputation can enhance performance in food SCQM [173]. Recent literature offers insight into how blockchain can support food SCQM, such as trust and reputation through traceability and supplier engagement, sustainability, improved monitoring and control, and provenance and authentication [68].

## 6 Discussion and areas of future work

### 6.1 Discussion

The discussion is below on three main points: i) Fit for Purpose SCQM Practices in AFNs, ii) Transferable Learning, and iii) Exploiting Advances in Digital Technologies for Improving SCQM Practices.

i) Fit for Purpose SCQM Practices in AFNs:

Practices should be designed to support the stakeholders in AFNs and not oppose them. AFNs should implement achievable SCQM practices or those that are realistic in the supply chain. Complex and top-down SCQM practices may be challenging for AFN stakeholders to adopt, and it is essential that such practices encompass the needs of stakeholders and support performance. An important finding is a need for geographical indication related to origin and provenance. This may be supported through quality governance schemes such as PDO (product designation of origin) and PGI (product of geographical indication) [100,101]. Other schemes found within the study relate to organic production [146], and quality management systems (IFS, BRC, ISO, GLOBALGAP) [134]. These schemes and associated labels can reinforce stakeholders' competitiveness in global food systems. However, the schemes may play a minor role in more locally based, face-to-face, and proximate supply chains in AFNs, which rely more on trust and less on labels. These more local and short food supply chains can be supported through trust-based, flexible governance systems and transparency [86]. Participatory guarantee systems can be used in these AFNs as a substitute for structural assurances. The participatory guaranteed systems may adopt a labelling approach. However, this could lead to problems associated with traditional quality labels, such as a need for more consumer understanding [174], reflecting on the importance of maintaining local knowledge in AFNs [175]. Digital technologies could instead be used to enable the trust to govern and provide information to consumers regarding the key practices in the supply chains of AFNs [68,176]. The primary considerations for fit-for-purpose SCQM practices in AFNs, derived from the systematic literature review, are in Table 8.

ii) Transferable Learning:

**Table 8:** Fit-for-purpose SCQM practices in AFNs.

Description	Fit for Purpose Description
Objective of practices	Practices that support AFNs to meet objectives (i.e. market performance, sales), as opposed to those that may impose barriers, i.e. labelling.
Practices design	Practices should be built in a democratic, open, and customisable way to meet the needs of AFNs while also considering the legal and market requirements in which they operate.
Traceability and transparency	Upstream practices should focus on the geographical origin of production and provenance. Traceability is critical, providing information on the supplier's quality on the production quality of inputs and final goods, production quality, process quality, local supply, supplier knowledge, and reputation management.
Governance and quality control	The governance of quality practices in AFNs is needed to provide assurances over aspects such as the geographical indication of origin. Structural assurances may be used (ISO, BRC. IFS), but are often difficult to obtain for SMEs, and therefore the use of more flexible and participatory governance systems may be beneficial.
Consumer Driven	AFNs are consumer-driven. Consumers demand high levels of food quality and safety related to a product, which should be evident to the consumer. Thus, communicating with the customers over upstream practices is critical. Supply-driven practices that large and industrialised players mainly define may be helpful through transferable learnings; however, they can also oppose the quality conventions of AFNs.
Information flow and digital technologies	Adopting digital technologies is helpful in AFNs, and technologies that support information flow and sharing are suggested, enabling transparency and traceability needs.
Trust and supply chain relationships	Trust and supply chain relationships between stakeholders are of high importance. Direct relationships, higher levels of cooperation/collaboration, transparency, and supply chain integration often support this. Arm's length and contrasting relationships within AFNs are undesirable. The SCQM practices in AFNs should focus on developing trust and relationships across the supply chain, thus moving towards more direct channels. Where face-to-face channels are not possible, enabling technologies can be beneficial.

The main SCQM practice categories in global food supply chains also widely apply to AFNs. Several differences include the merging of logistics and process quality management into internal quality management, customer service, and customer-focused relationships [35,58,59]. Trust, governance [177], and postponement [178] have yet to be recognised as main food SCQM practice categories in global food systems, but may be useful in AFNs. Human resource management, top management leadership and commitment, and continuous improvement have minor connections to AFNs. However, they may become more relevant as these supply chains develop. Some of the sub-category SCQM practices are more specific to AFNs, such as geographical indication (origin) of production/provenance and direct relationships. Other practices, for example, supply chain integration and adoption of enabling technologies, coexist with the supply chain in global food systems. The coexistence of practices offers an opportunity for transferable learning. Large food producers involved in the supply chains of globalised food systems have developed much experience in food production and are well prepared to meet downstream quality needs [133]. However, economic barriers can lead to challenges for SMEs in the AFN in meeting set standards [29,90].

SCQM practices could be facilitated by resource and information sharing and adopting digital technologies [99,133]. Product quality, product safety, process quality, and logistics quality are established in the food SCQM in mainstream supply chain networks in global food systems. Learning from these can be beneficial to AFN through SCQM practices like standardisation of production processes [99], quality monitoring [104], and setting quality criteria [115]. The quality of information and information systems are also well-developed in the supply chains of global food systems and have offerings to support those in AFNs. For example, AFNs can learn from information transparency and improve levels of traceability. These elements can support the traceability of internal and external processes and transparency for supply chain integration and internal quality management within AFNs. Also, trust has been identified as an essential factor across supply chains in global and AFNs. However, the practice of trust may not be transferable due to differences across food systems. For example, in face-to-face and proximate AFNs, localness and direct relationships are core constructs for trust in quality [138,143,147]. However, the extended AFN, where trust is established through quality schemes and labels, may learn from global food systems [129].

Review studies on SCQM practices in global food supply chains did not identify the product to meet seasonality constraints. However, this is an essential constraint for AFNs, particularly those using reduced proximity strategies, as production is limited to local conditions [136,179]. In addition, this study is an early identifier of provenance/geographical indication for an SCQM practice and thus may not benefit from transferable learning. Provenance/geographical indication reflects the desire of consumers in AFNs to know better the producer and origin of food [145].

iii) Exploiting Advances in Digital Technologies for Improving SCQM Practices:

Digital technologies are emerging that can support AFNs, for example, AI (Artificial intelligence), Big Data, Blockchain technology and IoT (Internet of Things) [142]. Blockchain and IoT show more short-term potential for the supply chains of AFNs to support fresh and organic food supply chains [180,181] and to protect the authenticity of local foods [182]. Currently, the data required for big data technology and AI technologies may be limited in the supply chains of AFNs and may require more sophisticated food networks. Therefore, these technologies may become more relevant as AFNs mature.

[98] present a blockchain-based system to enhance traceability and quality within a local food supply chain, showing some significant benefits through trustworthy information and transparency. Within these local food systems, blockchain can enhance local embeddedness and support rural development [183]. Blockchain helps improve performance through quicker access to information for better decision-making. Blockchain is also shown in fresh food supply chains [99], which suggests a positive effect on quality, integration, supply chain collaboration, and traceability in the fresh food supply chain [124]. [140] investigate the role of blockchain in delivery and distribution management, showing promising applications such as enhanced condition tracing over the supply chain. Other blockchain applications supporting SCQM in AFNs are shown in Refs. [141,148], highlighting significant abilities in traceability, transparency, and governance, such as upholding quality, linking to the origin, and reducing fraud in the food supply chain. Blockchain offerings for food supply chains are promising and can further benefit through system integration with other emerging technologies [184]. A significant benefit of blockchain in food supply chains is creating more direct links between producers and buyers, thus increasing competitiveness [185]. IoT is another promising technology. For example [126], explores the use of biomarkers in the context of IoT to provide a solution to the digital-physical boundary. The research suggests that biomarkers can improve visibility in the chain and help understand the SCQM practices of upstream supply chain members (i.e. producers). IoT has also been identified to support urban agricultural chains' fresh food supply chain [186], supporting quality and safety through a data-driven system. More current application of digital technologies in AFNs is the use of e-commerce platforms to facilitate transactions directly between consumers and producers [187].

### 6.2 Implications for theory and practices

This research has identified the SCQM practices within AFNs. Such practices can enhance supply chain quality management in AFNs, providing insight to assess the practices further. The proposed framework offers insight into how SCQM practices and quality conventions in AFNs can be linked, emphasising the importance of traceability, governance, provenance, transparency, trust, and supply chain relationships in AFNs. Based on supply chain and performance needs, stakeholders in AFNs may tailor SCQM practices to meet set objectives. Like supply chains in global food systems, formalised contracts and traceability are necessary to ensure transparency, trust, food quality, and safety. However, the development and implementation of these practices may vary across different supply chains. Theoretical and practical implications are highlighted below.

Theoretical implications.

1. The SCQM practices identified for AFNs can be used as a basis for further analysis and development to advance SCQM within the networks.
2. The proposed framework in Fig. 8 that links the SCQM practices and the AFN quality conventions can be used to assess the relevance of SCQM practices in AFNs through a stakeholder perspective.
3. There is potential for innovative governance structures in AFNs to support SCQM practices.

Practical implications.

1. The practices identified can serve as a basis for managers to gain an understanding of how implementing their chosen practices can help to improve SCQM performance in their AFNs and create opportunities for better governance, collaboration, and trust between stakeholders.
2. The SCQM practices should be tailored to each quadrant of the proposed framework in Fig. 8 and the specific needs of an AFN.
3. There is a need for clear and strict standards for governance and sanctions for non-compliance.
4. Digitalisation can support SCQM practices in AFNs, and it is important to understand the stakeholder requirements when developing and implementing such systems.
5. Top management support is required to ensure that practices adopted by their supply chain are fit for purpose and are appropriately implemented.

Supply chains in AFNs are responding to a drive for more sustainable and high-quality food supply chains (quality turn). Collaboration and innovation are crucial to sustainability performance in supply chains [188]. To meet sustainability objectives and quality needs, AFN stakeholders should recognise and implement areas to optimise their supply chains through digital technology, supporting more efficient supply chain processes for environmental performance [53]. Geographical indication and quality assurance-related practices can act as a means to improve sustainability in AFN supply chains. However, challenging practices such as transparency, governance, and traceability must be addressed to reach desired practice performance levels. Labelling practices can support the communication of sustainability and SCQM performance but can also cause information overload [129,174,189]. Ultimately, it is essential to recognise that different supply chains in AFNs can have varying outcomes relating to sustainability: Economic, environmental, and social [38]. Linking practices to the quality conventions can guide practitioners in AFNs in aligning their SCQM practices to their sustainability-related performance objectives.

### 6.3 Gaps and areas for future work

Supply chain quality management practices in AFNs have received little attention, as the focus has mainly been on quality conventions. A SCQM practices approach to support AFNs may be essential to enhance performance and ensure quality is upheld in the supply chain. It remains unclear, how the food SCQM practices put forward in the literature can be adapted to leverage the performance of supply chains of AFNs. There needs to be more effort to assess SCQM practices challenges, i.e. the areas in that may significantly impact SCQM practice performance in the AFNs. Establishing a method for identifying these challenges may be beneficial as these AFNs develop. Socio-technical constructs, such as total quality management culture, governance systems, and digital technologies, have been alluded to as influencing factors in food SCQM performance and improved market performance [133]. However, the hypothesis that these constructs influence SCQM practices in AFNs and overall performance has yet to be researched and evaluated. Precisely, the areas for future work are provided in Table 9.

**Table 9:** Gaps and areas of future work.

Motivation	Gaps	Areas of Future Work
Autocratic and institutionalised practices defined from the top down may not fit the unique needs of AFNs and act as a barrier to stakeholders.	The extent to which the SCQM practices are relevant for AFNs and the extent to which they may support SCQM performance still need to be studied.	Research on the SCQM practices on real-life AFNs through case studies and empirical investigation.
	The antecedents and challenges of SCQM practices in AFNs have not yet been investigated, and it is unclear how these antecedents and challenges have been defined.	Studies involving experts and stakeholders in AFNs to develop an understanding of the factors that may contribute to practice performance.
The information requirements for transparency and traceability needs may differ per supply chain. These requirements vary compared to those in the supply chains of industrialised food systems.	Identifying and analysing the information requirements for AFNs supporting these consumer-driven supply chains has not yet been investigated.	A consumer-focused study using a survey approach to understand the information requirements SCQM and transparency in AFNs.
Quality control systems should not act as a barrier to the initiatives in AFNs but instead support them. Barriers could be strict standards that are unnecessary for the market, high costs of adoption and a complex process to become certified and receive market access [29]. It is, therefore, necessary to understand the benefits and challenges of alternative governance systems in AFNs, for example, the PGS.	The alignment of quality governance systems to support SCQM in AFNs is unclear. It is, therefore, necessary to understand the benefits and challenges of alternative governance systems in AFNs, for example, the PGS.	Studies on the overreaching concept of quality governance and how it can support the definition of quality standards, quality assurance of compliance with the quality conventions and industry standards, and continuous quality improvement.
Due to the scale of AFNs and the companies residing within them, the information technology to support them may differ.	Digital technology is promising for supporting the governance of SCQM practices in AFNs; however, the system requirements still need to be studied. In addition, identifying the potential technologies to support information, transparency, and traceability in AFNs can be beneficial and is currently limited in research.	Studies on blockchain-based supply chain quality management in AFNs, including pre-adoption, adoption, and post-adoption considerations.
Global supply chains have shown to have benefited from transparency-enabling technology, i.e. blockchain. Also, its application and challenges are well developed in the supply chains in global food systems, however less so for those in AFNs.	Using digital technology to support transparency and information sharing in the supply chains on AFNS may benefit the actors. Studies on information needs and digital tools to support the information needed in the supply chains of AFNs would be beneficial.	Studies on information needs, the supply chain of AFNs and the use of digital technologies to support transparency.

Based on the research gaps and areas for future work, four main themes are identified: i) Factors contributing to practice performance, ii) Stakeholder studies on information requirements and transparency, iii) Exploring the use of digital technologies for SCQM in AFNs, and iv) Governance to support performance in the supply chains of AFNs.

## 7 Conclusion

This research used a systematic literature review methodology to identify supply chain quality management practices in sustainable food networks, with a focus on supply chains in alternative food networks (AFNs). The concept of quality is central to AFNs and is primarily based on norms, standards, and values that are driven by consumers. Findings of the review include that: 1) There are identifiable SCQM practices in AFNs contained in the literature, and opportunities exist in consolidating the practices through learning from established practices in supply chains of global food systems. Identified SCQM practices from the AFN literature include those associated with a) the need for provenance and geographical identification of production in supplier quality management, b) the need for supply chain relationships, c) the need for inclusion of trust, traceability, and transparency as core practices, and e) the need for governance; 2) SCQM practices can support the quality conventions of AFNs through improved links to place, producer, production, and (bio)processes; 3) The consumer is a crucial driver for quality in the AFN, thus reflecting orientations towards consumer-driven supply chain. Meeting customers' needs is critical in AFNs, and it is important for the supply chains to place increasing emphasis on appropriate fit-for-purpose downstream SCQM practices; 4) Governance is relevant for SCQM practices in AFNs and could be supported through more flexible and participatory quality systems. The use of local quality management systems is also relevant for quality governance; 5) Internal quality management practices like those associated with process and logistics quality management should be adapted to include a supply chain-inclusive strategy in order to enhance the AFN processes and traceability. Identifying practices can be beneficial to both researchers and practitioners in supply chains in sustainable food networks. The dynamics of the networks would likely require that practices need to be adapted and new ones created when necessary.

Further research is required to regularly refine, analyse, and assess the importance of SCQM practices in AFNs, especially through stakeholders' perspectives. The theoretical, review-based approach adopted for this study is a limitation. Although the literature provides insight into SCQM practices for AFNs, it still needs to be determined how the food SCQM practices put forward in the literature can be adapted to leverage those of AFNs currently used in practice. A key limitation of the systematic literature review is that although it allows the identification of practices, it needs to provide detailed insight into how those practices apply in real-world scenarios. In addition, the quality of papers included in the review is important. To validate, generalise and further develop the findings in this paper, it could be beneficial to utilise additional review methods such as meta-analysis, bibliometric analysis, and use of mixed methods. Several additional areas for future reviews were suggested, and they include in-depth reviews on the role of governance, transparency, information sharing, and digital technologies in relation to SCQM practices in sustainable food networks.

## CRediT authorship contribution statement

**Patrick Robert Burgess:** Conceptualization, Data curation, Formal analysis, Investigation, Methodology, Writing – original draft, Writing – review & editing. **Funlade T. Sunmola:** Conceptualization, Investigation, Methodology, Supervision, Validation, Writing – review & editing. **Sigrid Wertheim-Heck:** Conceptualization, Supervision, Writing – review & editing.

## Declaration of competing interest

The authors declare that they have no known competing financial interests or personal relationships that could have appeared to influence the work reported in this paper.
